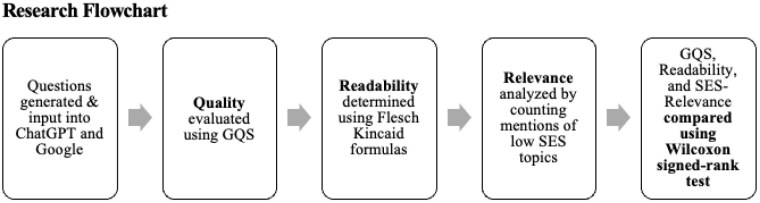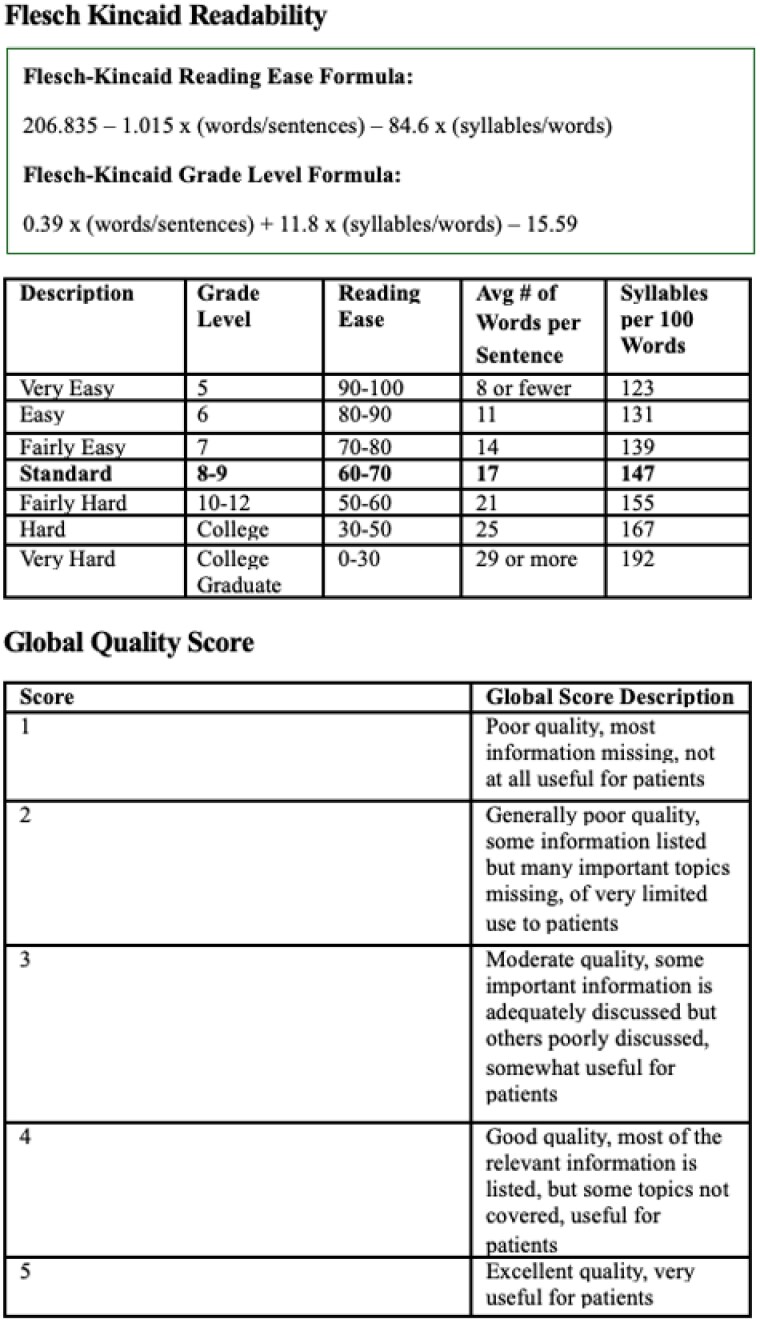# 605 Evaluating ChatGPT’s Utility in Addressing Socioeconomic Disparities in Burn Patients: A Comparative Study with Google

**DOI:** 10.1093/jbcr/iraf019.234

**Published:** 2025-04-01

**Authors:** Blancheneige Beohon, Joshua Lewis, Philong Nguyen, Matthew Dao, Mbinui Ghogomu, Steven Wolf, Amina El Ayadi, Juquan Song

**Affiliations:** University of Texas Medical Branch; University of Texas Medical Branch; University of Texas Medical Branch; University of Texas Medical Branch; University of Texas Medical Branch; University of Texas Medical Branch; University of Texas Medical Branch; University of Texas Medical Branch

## Abstract

**Introduction:**

Patients from low socioeconomic status (SES) backgrounds often face significant barriers to quality burn care, including limited healthcare access, follow-up, and health literacy. Many rely on online resources like Google for medical information, which can be overwhelming and lack relevance to their specific needs. This study compares the quality, accessibility, and SES-relevance of burn care information provided by ChatGPT and Google to address information disparities for low SES patients.

**Methods:**

A standardized set of commonly asked questions on immediate burn care, medical treatments, and long-term care was developed based on clinical guidelines. These questions were input into ChatGPT (version 4.0) and Google, with the first organic Google search result analyzed. For example, a question on immediate care asked, “How do I treat a burn at home?” Two medical students and two burn surgeons with over 15 years of experience independently assessed response accuracy using the Global Quality Score (GQS) on a scale of 1 (poor) to 5 (excellent). Readability was measured using the Flesch-Kincaid grade level, and SES-relevance was determined by counting responses addressing affordable treatments and access to care. A Wilcoxon signed-rank test was used to compare GQS, readability, and SES-relevance between ChatGPT and Google.

**Results:**

ChatGPT provided significantly higher-quality responses than Google, with an average GQS of 4.35 ± 0.60 versus 2.25 ± 1.10 for Google (p < 0.01). For half of the questions, respondents unanimously preferred ChatGPT for patient information. For the remaining five, two surgeons preferred ChatGPT, while one preferred Google. Both platforms fell within grade levels 8-9. ChatGPT addressed SES-related issues in 74% of its responses, while Google did so in 33%.

**Conclusions:**

ChatGPT outperformed Google in providing accurate and SES-relevant burn care information. These findings suggest that AI tools like ChatGPT could reduce health information disparities for low SES patients by offering tailored and user-friendly guidance. Future studies should validate these findings across other clinical topics and patient populations.

**Applicability of Research to Practice:**

This study highlights the potential of integrating AI tools like ChatGPT into patient education, particularly in underserved communities. By providing tailored, accessible, and SES-relevant information, AI tools could improve health literacy and outcomes for low SES patients. Further research should explore AI use in clinical settings and its impact on health disparities.

**Funding for the Study:**

N/A